# Practical Human Anatomy

**Published:** 1886-03

**Authors:** 


**Practical Human Anatomy.** A Working-Guide for Students of Medicine and a Ready Reference for Surgeons and Physicians. By FANEUIL D. WEISSE, M.D., Prof. of Practical and Surgical Anatomy, Medical Department of the University of the City of New York; Prof. of Anatomy, New York College of Dentistry; Prosector, 1863 to 1865, to the late Valentine Mott, M. D., LL. D.; Emeritus, Prof. of Surgery and Surgical Anatomy, Medical Department of the University of the City of New York. Illustrated by 222 Lettered Plates, containing 321 Figures. New York: William Wood & Co., 56 and 58 Lafayette Place. 1886.

After nearly twenty years' practical experience, Dr. Weisse gives his former students and the profession the benefit of his vast research. In the outset, we can say that no student of medicine, no surgeon, no physician, can afford to be without this work. If we were able to have but one anatomy in our library, this would be the one we would choose. The plan of the work embraces the following points: "1st, the division of the body into *practical* dissections; 2d, the giving, in *dissection paragraphs*, the progressive steps by which the several parts involved in a dissection are to be systematically displayed; 3d, the guidance by lines across the parts in the plates—called *section lines*—to the points where they are to be cut, for their reflection, in order to advance to a succeeding stage of the dissection; 4th, the indication, by *numbering the parts of the dissections*, of the order in which they are exposed; 5th, the description of the parts, in *descriptive anatomy paragraphs*, as they are brought into view; 6th, the adherence in expressing the relations of parts to a well-defined nomenclature of *general and special anatomical terms*; 7th, the illustration of the anatomy of the regions and viscera of the body by plates, *with the names of the parts printed upon them, or at the sides of the figures, with indicating lines to them*—the *dead-anatomy* is thus presented to the student, and the *living-anatomy* to the surgeon and physician." This was the Doctor's plan, and how faithfully he has carried it out the work bears witness. The lack of a well-defined nomenclature, or poor and ill-defined illustrations, has been the fault and weakness of some of our works on anatomy, but in this we can find neither poor illustrations nor bad nomenclature; both are excellent. "Too much cannot be said in praise of the comparatively wonderful artistic skill displayed by Mr. Maximillian Cohn, in his faithful reproductions of nature, given us in the plates and plate figures, and the clearness of his lettering of the same. The illustrations are photographic in their representations of nature, and are works of art in themselves." This quotation from the authors' preface is but just praise of the illustrations. They are all good; those of the brain are especially beautiful and clear. "Nature has been the *text*-book to which reference has always been made; but, a due respect for the labors of our fathers and of our contemporaries has been kept in view." The work is one of the best which has been offered to the profession. The excellent character of the work of the publishers has added much to it, and it is bringing them, from all directions, well-deserved praise.

